# Putting the *t* in *tools*: a roadmap for implementation of new global and regional transgender guidance

**DOI:** 10.7448/IAS.19.3.20801

**Published:** 2016-07-17

**Authors:** R Cameron Wolf, Darrin Adams, Robyn Dayton, Annette Verster, Joe Wong, Marcela Romero, Rafael Mazin, Edmund Settle, Tim Sladden, JoAnne Keatley

**Affiliations:** 1 Office of HIV/AIDS, United States Agency for International Development, Washington, DC, USA; 2 Key Populations Program, Center for Public Health and Human Rights, Department of Epidemiology, Johns Hopkins Bloomberg School of Public Health, Baltimore, MD, USA; 3 Linkages across the Continuum of HIV Services for Key Populations, FHI 360 Durham, NC, USA; 4 Department of HIV/AIDS, World Health Organization, Geneva, Switzerland; 5 Asia Pacific Transgender Network, Bangkok, Thailand; 6 REDLACTRANS, Buenos Aires, Argentina; 7 Pan American Health Organization, Washington, DC, USA; 8 United Nations Development Programme, Bangkok, Thailand; 9 United Nations Population Fund, New York, NY, USA; 10 Center of Excellence for Transgender Health, University of California San Francisco, CA, USA

**Keywords:** Trans, transgender, HIV, regional blueprint, tools, TRANSIT, MSM

## Abstract

Transgender (trans) activists and global health partners have collaborated to develop new tools and guidance for assessing and addressing HIV and other health needs within trans populations. Trans women experience a heavy burden of HIV and other sexually transmitted infections (STIs), high incidence of violence and difficulties accessing gender-affirming services. At the same time, little has been published on trans men's health, HIV issues, needs and experiences. Young trans people are especially marginalized and vulnerable, with few programmes and services specifically tailored to their needs. Trans-specific data and guidance are needed to adapt the global response to HIV to meet the needs of the trans population. While the needs of this group have only recently received attention, global, regional and other technical guidance documents are being developed to address these gaps. Regional blueprints for comprehensive care for trans people in Latin America, the Caribbean, and Asia and the Pacific are now available. These tools – supported by the Pan American Health Organization, World Health Organization, US President's Emergency Plan for AIDS Relief and the United Nations Development Programme, in collaboration with regional trans groups – provide a contextual map, indicating opportunities for interventions in health, HIV, violence, stigma and discrimination, social protection and human rights. Global guidance includes the World Health Organization's *Policy Brief: Transgender People and HIV*, and the interagency publication, *Implementing Comprehensive HIV and STI Programmes with Transgender People*. Community empowerment and capacity building are the focus of the new tools for global and regional transgender guidance. The goal is to strengthen and ensure community-led responses to the HIV challenge in trans populations. This article describes the new tools and guidance and considers the steps needed to use them to appropriately support and engage transgender populations within national AIDS, STI, and sexual and reproductive health responses and programmes. The time to use these tools and guidance for advocacy, strategic planning, capacity building, programme design and training is now.

## Introduction

In just the past five years, the global health community has gone from almost no mention of transgender health and rights to an awakening recognition of the importance of addressing transgender (trans) people as a key population independent of men who have sex with men (MSM). Although some countries supported trans programming through the Global Fund's 2010 *Strategy in Relation to Sexual Orientation and Gender Identitie*s [1], a shift occurred when the findings from the first HIV prevalence meta-analysis on trans women became available. Presented at the 2012 International AIDS Conference in Washington, DC, the results showed that transgender women were 49 times more likely to acquire HIV than the general population – a finding that, along with strong personal statements from noted trans activists, finally garnered global recognition among public health professionals [2,3]. That same year, the Global Commission on HIV and the Law published *HIV and the Law: Risks, Rights & Health*, which provided specific recommendations to ensure access to equitable healthcare for trans people [4]. In 2013, the US President's Emergency Plan for AIDS Relief (PEPFAR) designated transgender persons as a key population independent of MSM in the development of the *Technical Considerations for Country Operational Plan* [5]. Then, in 2014, the WHO included separate recommendations for trans populations within its *Consolidated Guidelines on HIV Prevention, Diagnosis, Treatment and Care for Key Populations* [6], as it did again in the 2015 supplement, *Tool to Set and Monitor Targets for HIV Prevention, Diagnosis, Treatment and Care for Key Populations* [7].

This change was long overdue. Trans women experience a heavy burden of HIV and other sexually transmitted infections (STIs) [2], high incidence of violence [8–11] and difficulties accessing and being retained in gender-affirming services [12,13]. The available literature has little material on trans men's health, HIV issues, needs and experiences [14–17]. Young trans people and trans sex workers are especially marginalized and vulnerable, with few programmes and services specifically tailored for their needs [18–26].

While recognition of the need to focus increased attention on the trans community took far too long, tools to inform programmes and policies that better serve trans people have been quickly developed. In 2016, we not only have the support documents described above, we have a set of global and regional tools that offer guidance for assessing and addressing HIV and other health needs in trans communities. Even more important than the fact of their existence, the process of creating these tools set a new standard for collaboration between trans activists and global development partners. The resulting products were informed by trans expertise and experience and can be equally owned and implemented by donors, programme managers, activists and trans community members. In this article, we present this set of complementary tools, describe the process of their development and call for their broad and immediate implementation.

## Discussion

### Regional tools for Latin America, the Caribbean, and Asia and the Pacific

Three *regional blueprints* for meeting the comprehensive health and rights needs of transgender people were recently developed [8,27,28]. All were designed to address the regional needs and diversity within trans communities and were developed under the leadership of trans people in collaboration with development partners. The blueprints describe the broader, holistic needs of trans people and cover topics including gender affirmation in all forms of trans-competent care, transition, HIV-related guidance, social protection and human rights. The regional blueprints are meant for *guidance*, *adaptation* and *translation* (not adoption). For example, they include medical algorithms from expert clinical guidance but do not specify recommended dosages or drug names [29,30]. They are designed to inform and be used by a wide range of stakeholders, including trans community members, advocates, public-health practitioners, clinicians, donors and governments.

The regional *Por la Salud de las Personas Trans: Elementos para el desarrollo de la atención integral de personas trans y sus comunidades en Latinoamérica y el Caribe* (‘Blueprint for the Provision of Comprehensive Care for Trans Persons and Their Communities in Latin America and the Caribbean’) (2013) [27] and the *Blueprint for the Provision of Comprehensive Care for Trans Persons and Their Communities in the Caribbean and Other Anglophone Countries* (2014) [8] were conceived at the same time. The Pan American Health Organization hired two writers – one of them a member of the trans community – to lead the creation of a document for policymakers and healthcare professionals. The writers developed a framework that was reviewed and revised by trans activists and health experts at an initial meeting. Additional consultations were planned to capture the day-to-day needs of trans people throughout the region.

During consultation planning it was determined that two blueprints were needed to be responsive to the differences between Latin America and the Caribbean. For the Latin American blueprint, consultations were held in Central America, South America and Mexico with trans community members, non-governmental organizations and donors, and representatives of national ministries of health (MOHs). The participants clarified terminology, developed a list of regionally appropriate definitions and highlighted priority medical topics. As trans ownership increased, community members began to talk about the document as their own and the audience and intention of the document shifted. The end result was an advocacy and educational tool that could also be used by members of the trans community. Once the Latin American blueprint was complete, it was translated to English and Caribbean trans community members and other stakeholders provided feedback during a meeting in Trinidad. As a result of their input, the completed Caribbean document acknowledges the more restrictive legal environment and includes local case studies.

The Latin America and Caribbean (LAC) regional blueprints have been widely used. Trans activists affiliated with REDLACTRANS and involved in the blueprints’ development used the documents and the process of their creation to advocate to and partner with MOHs in Bolivia, Guatemala, El Salvador, Peru and Argentina. In each country, MOH officials are now training healthcare providers to offer trans-competent services. Discussions with health officials in Brazil, Trinidad and Tobago, and the Dominican Republic are ongoing. In Argentina, the Latin America blueprint was adapted for national needs and re-branded [31]. At the regional level, the blueprints form the basis of culturally and clinically appropriate guidance in both Latin America [32] and the Caribbean [33].

The *Blueprint for the Provision of Comprehensive Care for Trans Persons and Trans Communities in Asia and the Pacific* (2015) [28] was built on the model of the LAC blueprints but took unique shape based on the diverse needs and context of trans people in the region. As with the other regional blueprints, its focus is strengthening health and human rights responses. It emphasizes gender identity recognition and the provision of gender-affirming health services and fills a gap in information on medical transitioning.

As in LAC, trans leadership was important from the inception of the Asia and the Pacific regional blueprint. The Asia Pacific Transgender Network (APTN) was the key regional community partner in collaboration with the United Nations Development Programme (through the Multi-Country South Asia Global Fund HIV Programme and the Being LGBT in Asia initiative) and the PEPFAR/USAID-funded Health Policy Project. APTN facilitated the participation of trans people from all over Asia and the Pacific, people with diverse languages, cultures, histories and identities [34] who were actively involved in trans health and rights promotion. A trans man authored and led the process of developing the blueprint, which allowed the document to describe important issues in the voice of the community. A trans woman from Asia coordinated efforts among the various stakeholders, including trans leaders and communities. The highly consultative process created ownership of the document and, even before completion, the blueprint was used for advocacy purposes. Furthermore, the blueprint has shaped local activists’ thinking on entry points for advancing human rights for trans people regionally and has created a platform for advocates, programmers, governments and donors to plan for implementation.

### Global tools

When the regional blueprints were conceived, there was no global guidance document specific to trans people and HIV. This situation has changed. Two trans-specific global tools have recently been developed: the World Health Organization's (WHO) *Policy Brief: Transgender People and HIV* (2015) [35] and *Implementing Comprehensive HIV and STI Programmes with Transgender People* (2016) [36]. Both were informed by the WHO's 2014 consolidated guidelines [6], including its annex, *Values and Preferences of Transgender People: A Qualitative Study* [12]. They were also influenced by the principles, development processes and content in the regional blueprints. These documents are more focused on HIV and transgender women, based on the epidemiology, than the regional blueprints. Both tools make note of the critical intersection between health access and human rights.

#### Policy Brief: Transgender People and HIV

This policy brief summarizes transgender-specific essential information and existing WHO recommendations, such as those in the consolidated guidelines. It was developed by WHO in collaboration with the International Reference Group on Trans People and HIV (IRGT). While the WHO has endorsed all the regional blueprints, the policy brief is the source for WHO-endorsed global trans-specific HIV guidance. It makes the case for serving the trans community, reminds decision makers that trans people have the right to health services and shares essential strategies. It is designed for policymakers and those conducting higher level advocacy, for example, with national governments. It directs readers to resource documents, such as the regional blueprints, for information on implementation.

#### Implementing Comprehensive HIV and STI Programmes with Transgender People (*Trans Implementation Tool* or *TransIT*)

This is one of a series of global implementation tools for key populations affected by HIV. Under development during 2014 and 2015, *TransIT* was published in early 2016. It provides how-to guidance on the delivery of HIV programmes with and for transgender people, particularly trans women who are disproportionately burdened with HIV. Taking the current best and promising practices in HIV programming for transgender people from the WHO consolidated guidelines and informed by the WHO policy brief, the implementation tool lays out practical approaches for setting up programmes and services that are built on a strong foundation of community engagement and empowerment. There is guidance on addressing stigma and discrimination; realizing and achieving human rights for trans people; delivering gender-affirming services that address HIV, other STIs, violence prevention and response, sexual and reproductive health needs, and co-infections and co-morbidities; service delivery approaches; and programme management.

*TransIT* was developed with input from many key partners and experts and with leadership from the trans community (including the IRGT), development partners, policymakers, public health practitioners and clinicians. Trans community members wrote portions of the tool and provided extensive feedback, which was obtained through a collaborative process and community consultation with participants from around the world.

Due to a lack of data and guidance on the AIDS epidemic among trans men and women, trans persons have been doing what marginalized people have done throughout history: they have been resilient [37,38]. They have comforted one another when faced with the deaths of their friends and loved ones; they have coped with violence in their communities; and they have fostered their own support networks in the midst of anger and isolation from family, religious groups and healthcare providers. They have raised their voices to the global silence, demanding action. The tools highlighted in this article are not just documents; they represent the voices of human beings who demand an equal right to health and well-being.

Although these tools represent an important step forward, there is more work to be done. LAC and Asia/Pacific countries lead regional efforts to address the health and human rights needs of trans people, but countries in the global North do not assign sufficient resources to this issue, as evidenced by poor health outcomes. Moreover, there is still “programming silence” in most of Africa, Central Asia and the Middle East. Given that local movements and networks may be nascent or non-existent and violence is an ever-present threat in these regions, it is important to use the new global tools to build the rationale with data and sensitization in these areas. This does not mean letting the situation lie dormant; it means engaging and including trans communities, building capacity and galvanizing advocacy efforts, including with funders, so that region-specific activities can be implemented. Given that sub-Saharan Africa is at the heart of the global HIV pandemic, future momentum should be focused there. In particular, advocates should point to the global HIV burden and human rights abuses, highlighting the connection between the two in the trans community.

### Advocacy

Regional networks and international trans groups already exist. In the process of addressing HIV, donors have convened trans advocates and country stakeholders to review the regional and global tools. This process must continue to evolve and flow into policy discussions at the country level. Most importantly, regional and country leaders should support and advocate for the inclusion of trans people as key populations in HIV national strategic plans and programmes supported by the Global Fund.

### Strategic planning and capacity building

Donors should help develop leadership and build the capacity of regional and national networks that will meaningfully introduce trans voices into strategic planning processes. Points of entry include the Global Fund Country Coordinating Mechanisms and the development of country operation plans by PEPFAR and other agencies. The new tools, including the WHO target setting and monitoring tool [7], provide a clear framework that communities and donors can use together for strategic planning processes linked to funding decisions.

### Programme design

Local case studies are highlighted, particularly in *TransIT* and the blueprints, and provide useful programme models that can be adopted through regional exchanges (i.e., south-to-south sharing) and technical capacity development via the regional and local networks.

### Training

All health facility staff need sensitization and training to develop clinical and cultural competency – topics covered in detail in the regional tools. For trans people, gender-affirming care is an effective link to HIV services [12,39], particularly because stigma, discrimination and violence may mean that HIV is not the first priority for trans people.

## Conclusions

The process of developing new tools for global and regional trans guidance has empowered leaders, blossomed networks and created new opportunities to focus our HIV prevention efforts for greater impact and epidemic control. In this new era of the UNAIDS 90-90-90 targets for HIV testing, treatment and care [40], we as trans community members, advocates, public-health practitioners, donors and governments must first reach trans people not yet engaged in programming and build trust. While we need better information, both on epidemiology and on the implementation of trans programmes, we cannot afford to wait for more data in order to act. We already know what to do. We need to focus on advocacy, strategic planning, capacity building, programme design and training. We need to strengthen and scale up the existing regional efforts in Asia, the Pacific and LAC. These programmes are ready to take steps toward improved quality and scale-up. Finally, we need to begin new efforts in other regions – offering trans-competent services while supporting existing and/or nascent trans organizations and movements. Now is the time for action.
